# Histone deacetylases repress the accumulation of licochalcone A by inhibiting the expression of flavonoid biosynthetic pathway-related genes in licorice (*Glycyrrhiza inflata*)

**DOI:** 10.1186/s43897-025-00144-4

**Published:** 2025-05-06

**Authors:** Jiangyi Zeng, Xiaoling Ma, Yuping Li, Lijun Zhou, Jingxian Fu, Hongxia Wang, Yongliang Liu, Ling Yuan, Ying Wang, Yongqing Li

**Affiliations:** 1 Guangdong Provincial Key Laboratory of Applied Botany, South China, Botanical Garden, Chinese Academy of Sciences, Guangzhou, 510650 China; 2 College of Life Science, Gannan Normal University, Ganzhou, Jiangxi 341000 People’s Republic of China; 3 University of Chinese Academy of Sciences, Beijing, 100049 China; 4 School of Traditional Chinese Medicine, Jiangxi University of Traditional Chinese Medicine, Nanchang, Jiangxi 330004 China; 5 Department of Basic Medical Science, Quanzhou Medical College, Quanzhou, Fujian 362011 China; 6 Shanghai Key Laboratory of Plant Functional Genomics and Resources, Shanghai Chenshan Botanical Garden, Shanghai, 201602 China; 7 Department of Plant and Soil Sciences, University of Kentucky, Lexington, KY 40506 USA; 8 Guangdong Provincial Key Laboratory of Digital Botanical Garden, South China Botanical Garden, Chinese Academy of Sciences, Guangzhou, 510650 China

**Keywords:** Histone deacetylase, Histone deacetylase inhibitor, Licochalcone A, Flavonoid, *Glycyrrhiza inflata* B.

## Abstract

Histone deacetylases (HDACs) play a crucial role in regulating plant growth, stress responses, and specialized metabolism. Licorice, utilized as both food and herbal medicine for millennia, includes *Glycyrrhiza inflata* as one of its primary medicinal species used globally. This study investigated the regulatory function of HDAC-mediated histone deacetylation in flavonoid biosynthesis in licorice. The research identified nineteen HDACs in the *G. inflata* genome. Abiotic stresses and plant hormones were found to influence flavonoid compound accumulation, correlating with altered expression patterns of *HDAC* genes and global histone H3 acetylation (H3ac) levels. Notably, several HDAC inhibitors enhanced flavonoid accumulation in *G. inflata*. Subsequent RNA-seq analysis revealed that the HDAC inhibitor suberoylanilide hydroxamic acid (SAHA) activated the expression of multiple genes related to flavonoid biosynthesis. ChIP-qPCR demonstrated that SAHA treatment increased the H3ac levels of flavonoid synthesis-related genes. Furthermore, overexpression of *GiHDA2b*, an *HDAC* member, decreased, while RNAi of *GiHDA2b* increased, the levels of expression and H3K18 acetylation of licochalcone A (LCA) biosynthetic genes indicating its negative role in flavonoid biosynthesis. This research provides valuable insights into the regulatory roles of *GiHDACs* and histone deacetylation in flavonoid biosynthesis in licorice, potentially contributing to improved bioactive compound production in medicinal plants.

**Supplementary Information:**

The online version contains supplementary material available at 10.1186/s43897-025-00144-4.

## Core

HDAC inhibitors enhance flavonoid production in *Glycyrrhiza inflata* by modulating histone acetylation levels and activating flavonoid biosynthesis-related gene expression. GiHDA2b, a HDAC member, negatively regulates flavonoid accumulation by decreasing the acetylation levels of H3K18 on the promoters of licochalcone A biosynthesis genes.

## Gene and accession numbers

The genome sequence of *G. inflata* utilized in this study was provided by our research group (https://ngdc.cncb.ac.cn/; NGDC; CRA009044; accessed on 25 November 2022). The *Arabidopsis* HDAC data were obtained from TAIR (https://www.arabidopsis.org/). The transcriptome data were deposited in the NCBI with the accession numbers PRJNA574093 and CRA011905.

## Introduction

Medicinal plants are highly valued in the healthcare and pharmaceutical industries due to their natural bioactive molecules (Maria et al. 2019). Licorice (*Glycyrrhizae Radix et Rhizoma*), a perennial herb of the *Fabaceae* family, is a widely used herb native to southern Europe and parts of Asia (Hosseinzadeh et al. 2015; Jiang et al. 2020). An integrative phylogenomics-MLA (machine learning analysis) method has identified over 10 species of licorice (Pastorino et al. 2018), with *G. uralensis*, *G. glabra*, and *G. inflata* being the primary medicinal species utilized (Commission et al. 2020). Licorice produces more than 400 bioactive compounds, including liquiritin, glycyrrhizin, and licochalcone, which exhibit a range of pharmacological effects, such as anti-inflammatory, anti-cancer, and antioxidant properties (Siracusa et al. 2011; Wei et al. 2015; Yan et al. 2023). These compounds are extensively used in the tobacco, food, and chemical industries. Additionally, each of the three licorice species possesses its own characteristic compounds. The chemical composition of *G. inflata* differs significantly from other commonly used licorice species, with its compounds being structurally related to trans-chalcone (Yang et al. 2018). Among these, licochalcone A (LCA) has the highest content and is the primary flavonoid monomer in *G. inflata*, making it a characteristic component of this species (Zhao et al. 2013). The elongated conjugated structure of LCA provides flexibility, contributing to its diverse biological effects, including significant antimicrobial and antioxidant properties. Due to its high potential economic value, LCA is widely applied in the medical and skincare industries (Chen et al. 2017). However, the LCA biosynthetic pathways and their molecular regulatory mechanisms remain largely undefined. To date, only an *O*-methyltransferase GiLMT1 has been demonstrated to be essential for LCA biosynthesis, potentially serving as a target of a histone deacetylase GiSRT2 (Zeng et al. 2023).

Both internal and external factors, including epigenetic modifications, play crucial roles in regulating the production of plant specialized metabolites (Hao and Xiao 2018; Li et al. 2022). Histone acetylation is a key factor in transcriptional activation (Castillo et al. 2017), which regulates multiple plant biological processes, including specialized metabolite biosynthesis (Chen et al. 2020), organogenesis (Chen et al. 2019; Deng et al. 2021), and stress responses (Kim et al. 2015; Li et al. 2019). Modulation of acetylation levels on H3K9 and H4K5 has been demonstrated to coordinate these processes (Li et al. 2014; Zhao et al. 2014a; Zhao et al. 2014b; Wang et al. 2015). The maintenance of histone acetylation homeostasis is regulated by the reciprocal interplay between histone deacetylases (HDACs) and histone acetyltransferases (HATs) (Yuan et al. 2020). In plants, HDACs are classified into three subfamilies based on their sequence similarity and co-factors: RPD3/HDA1, SIR2, and HD2. The RPD3/HDA1 subfamily is strongly associated with yeast and common in eukaryotes, while the HD2 subfamily is plant-specific (Hollender et al. 2008). All members of the RPD3/HDA1 subfamily possess a unique histone deacetylase domain and are ubiquitous throughout eukaryotes; HD2 is a plant-specific HDAC initially discovered in maize (Lusser et al. 1997). The SIR2 family (sirtuins) represents a distinct class of HDACs with no structural similarity to other types of HDACs, and the number of SIR2 family proteins identified in plants is relatively limited (Haigis et al. 2006).

HDAC inhibitors, including suberoylanilide hydroxamic acid (SAHA), trichostatin (TSA), sodium butyrate (NaB), and sirtinol (SIR), have been demonstrated to elevate the acetylation levels of histones and other target proteins, offering insights into the regulatory mechanism (Lu et al. 2021; Patanun et al. 2016). These inhibitors are categorized into distinct chemical classes and target various HDAC types. SAHA is the first HDAC inhibitor developed, while TSA, the initial natural oximate salt and HDAC inhibitor discovered, primarily targets class I and II HDACs. NaB predominantly affects class I and II HDACs, whereas SIR mainly influences class III HDACs (Dokmanovic et al. 2007). In plant research, HDAC inhibitors have been employed to examine the role of histone acetylation in developmental processes and stress responses. For instance, the introduction of HDAC inhibitors to plant cell cultures has resulted in increased levels of transgene expression and protein accumulation, indicating that HDAC inhibitors may enhance recombinant protein production in plant cell suspensions (Santos et al. 2018). Furthermore, in sea buckthorn (*Hippophae rhamnoides*), TSA treatment significantly down-regulates the expression of *HrHDA6*, *HrHDA9*, and *HrHDA19* genes during drought stress (Li et al. 2023).

HDACs are initially identified as histone-modifying enzymes regulating specialized metabolism in fungi. In *A. nidulans*, HDAC is reported as a negative regulator of sterigmatocystin and penicillin biosynthesis (Shwab et al. 2007). TSA treatment is found to affect the accumulation of specialized metabolites in both *Alternaria alternata* and *Penicillium expansum* (Shwab et al. 2007). Recent research has demonstrated significant progress in understanding the regulation of specialized metabolism by histone acetylation modification in plants. For instance, histone acetylation has been shown to influence the stress-induced formation of quality-related metabolites in tea (Gu et al. 2022), anthocyanin accumulation in Malus and citrus (Peng et al. 2020; Zhang et al. 2022), cannabinoid synthesis in hemp (Yang et al. 2021), and overall specialized metabolite biosynthesis in bamboo (Nomura et al. 2021). However, the role of HDACs in licorice remains largely unexplored. To date, only one HDAC in *G. inflata*, GiSRT2, has been reported to regulate flavonoid biosynthesis (Ding et al. 2012). A comprehensive analysis of the HDAC gene family across all licorice species is currently lacking.

This study investigated the regulatory role of HDAC-mediated histone deacetylation in flavonoid biosynthesis in licorice. Nineteen *HDAC* family members were identified in the *G. inflata* genome, and their subcellular localization and expression patterns were characterized. The research demonstrated that abiotic stresses and plant hormones influence flavonoid compound accumulation in *G. inflata*, correlating with altered *HDAC* gene expression patterns and global histone H3 acetylation (H3ac) levels. Additionally, HDAC inhibitors were found to promote flavonoid accumulation in *G. inflata*. RNA-seq and ChIP-qPCR analyses revealed that SAHA activated the expression of genes related to flavonoid biosynthesis and increased the H3ac levels of these genes. Furthermore, the study identified GiHDAC genes (*GiHDA2b*), an *HDAC* member, as a suppressor of genes involved in flavonoid synthesis. This research provides valuable insights into the regulatory roles of *GiHDACs* and histone deacetylation in flavonoid biosynthesis in licorice, potentially contributing to enhanced production of bioactive compounds in medicinal plants.

## Results

### HDAC inhibitor treatments enhanced flavonoid production in *G. inflata*

**Fig. 1:**
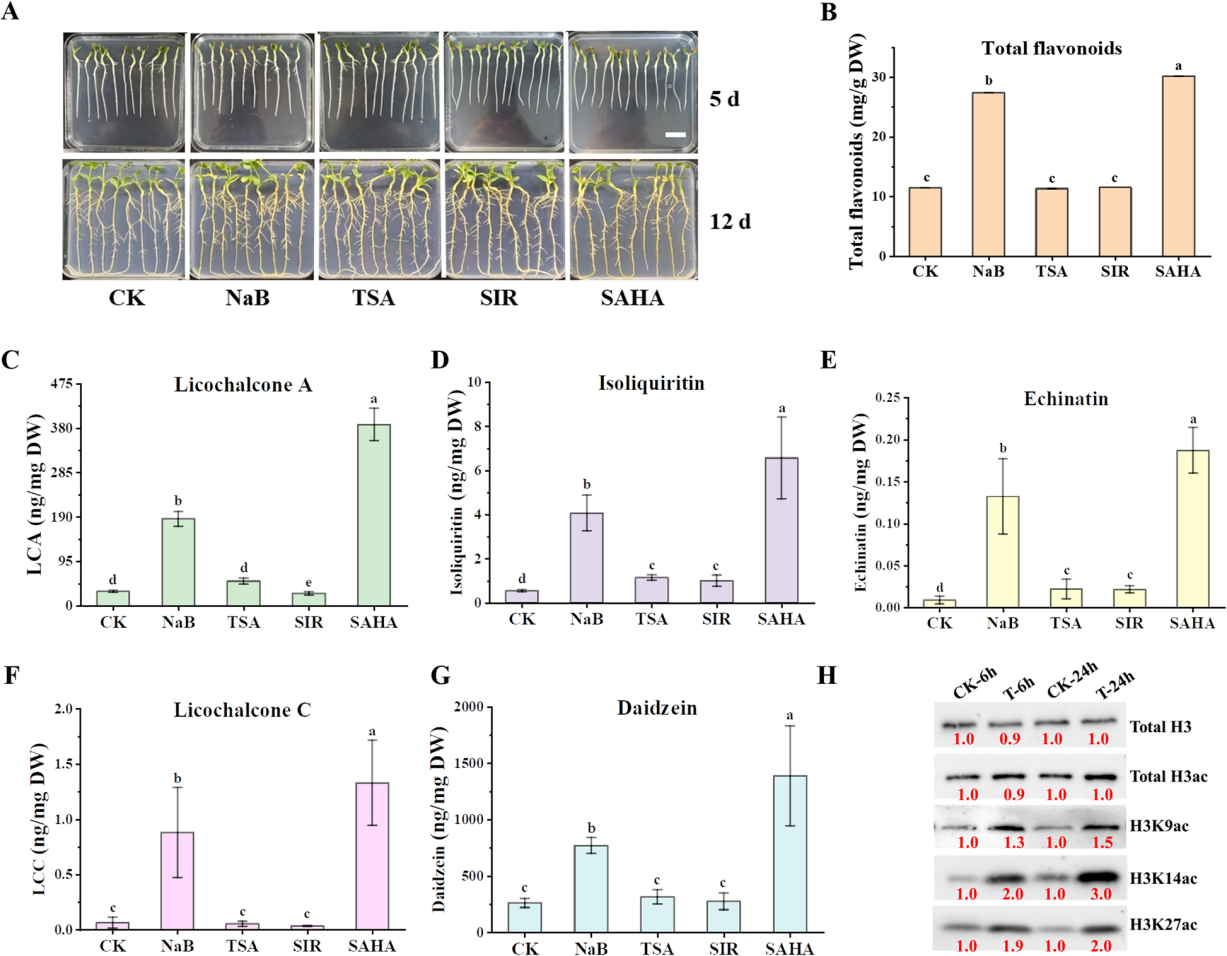
The enhancing effect of HDAC inhibitors on metabolite accumulation in *G. inflata*. **A** The phenotype of *G. inflata* seedlings treated with HDAC inhibitors. From left to right: 5-day-old *G. inflata* seedlings were treated with DMSO (Control), 1 mM NaB, 1 μM TSA, 10 μM SIR, and 100 μM SAHA for 7 days, respectively. Scale bar = 2.0 cm. The contents of total flavonoids (**B**), LCA (**C**), isoliquiritin (**D**), echinatin (**E**), licochalcone C (LCC) (**F**) and daidzein (**G**) in *G. inflata* seedlings were quantified by HPLC. Lowercase letters indicate significant differences at *P* < 0.05 among samples using Student's *t*-test, n ≥ 3. **H** Immunoblot analysis of histone in *G. inflata* seedlings treated with or without 100 μM SAHA for 6 and 24 h. ImageJ software was utilized to quantify the relative band intensities. Histone acetylation was detected using antibodies specific to H3ac, H3K9ac, H3K14ac, and H3K27ac, with histone H3 serving as the internal control for nuclear protein loading. The red numbers indicate the relative quantitative values of band intensity

To examine the influence of histone acetylation on the specialized metabolism of *G. inflata*, 7-day-old seedlings were exposed to various HDAC inhibitors: NaB (1 mM), TSA (1 µM), SIR (10 µM), or SAHA (100 µM) for 7 days. Control plants were treated with DMSO. The findings demonstrated a notable increase in total flavonoid accumulation following NaB treatment (Fig. 1B). In contrast, SAHA treatment showed a more pronounced enhancement of total flavonoid accumulation (Fig. 1B), but significantly impeded root growth, with fresh weight approximately 0.6 times that of the control (Fig. S1). TSA and SIR treatments had minimal effects on total flavonoid accumulation and plant growth (Fig. 1B; Fig. S1).

To further examine the effects of HDAC inhibitors on LCA accumulation, we quantified the levels of LCA, its precursor echinatin, its isomer licochalcone C (LCC), and other flavonoids including isoliquiritin and the isoflavone daidzein in licorice roots. Figure 1C-G demonstrates that plants treated with NaB and SAHA exhibited significantly higher concentrations of these compounds compared to the control group (CK). Given that SAHA demonstrated a more pronounced impact on LCA accumulation, our subsequent analyses focused on *G. inflata* plants treated with SAHA.

Histone H3 is recognized as the most extensively acetylated histone in plants, while histone H4 is the primary target of acetylation in animals and fungi (Lusser et al. 2001). We employed H3ac antibodies in western blotting to quantify histone acetylation in SAHA-treated samples (Fig.1H). The acetylation of histone H3 (total H3ac), including H3 lysine 9 (H3K9ac), H3 lysine 14 (H3K14ac), and H3 lysine 27 (H3K27ac), increased by 1.7, 1.3, 1.9, and 1.4 times, respectively, following 6 h of SAHA treatment (T-6 h) (Fig. 1H; Fig. S2; Fig. S3). When the treatment duration was extended to 24 h (T-24 h), the acetylation levels of H3ac and H3K27ac were similar to those observed at T-6 h, whereas H3K9ac and H3K14ac demonstrated elevated acetylation levels. These results indicate that SAHA, as an HDAC inhibitor, modulates histone acetylation on H3 in *G. inflata*.

### SAHA treatment activated flavonoid biosynthesis-related gene expression by modulating histone acetylation levels in their promoters

Based on the aforementioned findings, we hypothesize that elevated histone H3 acetylation levels significantly influence flavonoid biosynthesis. To examine this hypothesis, we conducted transcriptome sequencing analysis on SAHA-treated samples to comprehensively assess SAHA's impact on gene expression. RNA-seq analysis yielded 503 million high-quality reads (from 616 million raw reads), averaging 42 million reads per sample (Table S1). These high-quality reads were subsequently aligned to the *G. inflata* reference genome, achieving mapping rates of 93.71% to 94.53% across samples. Supplementary Table S1 provides summaries of raw sequence quality pre- and post-filtering, as well as the number of reads aligned to the reference genome. Principal component analysis (PCA) revealed substantial transcriptomic differences between SAHA-treated and untreated samples (Fig. S4A). Moreover, the correlation coefficient among the three biological replicates exceeded 0.995, indicating a strong intra-replicate correlation.

In the comparison group CK-6 h vs. T-6 h, 2781 differentially expressed genes (DEGs) were identified, comprising 1949 up-regulated and 832 down-regulated DEGs. The CK-24 h vs. T-24 h comparison group revealed 1559 up-regulated and 752 down-regulated genes (Fig. S4B-E). Among the DEGs, 1632 were unique to the CK-6 h vs. T-6 h group, 1162 were specific to the CK-24 h vs. T-24 h group, and 1149 were common to both groups (Fig. S4B). To verify the expression patterns of selected DEGs identified from RNA-seq, qRT-PCR was conducted on eight DEGs (Fig. S5), confirming the reliability of the RNA-seq results. KEGG enrichment analysis indicated that the up-regulated DEGs were primarily associated with specialized metabolite biosynthesis, including phenylpropanoid, diterpenoid, flavonoid, and isoflavonoid pathways. Furthermore, GO enrichment analysis revealed significant enrichment of metabolic processes among these DEGs (Fig. S6).

**Fig. 2:**
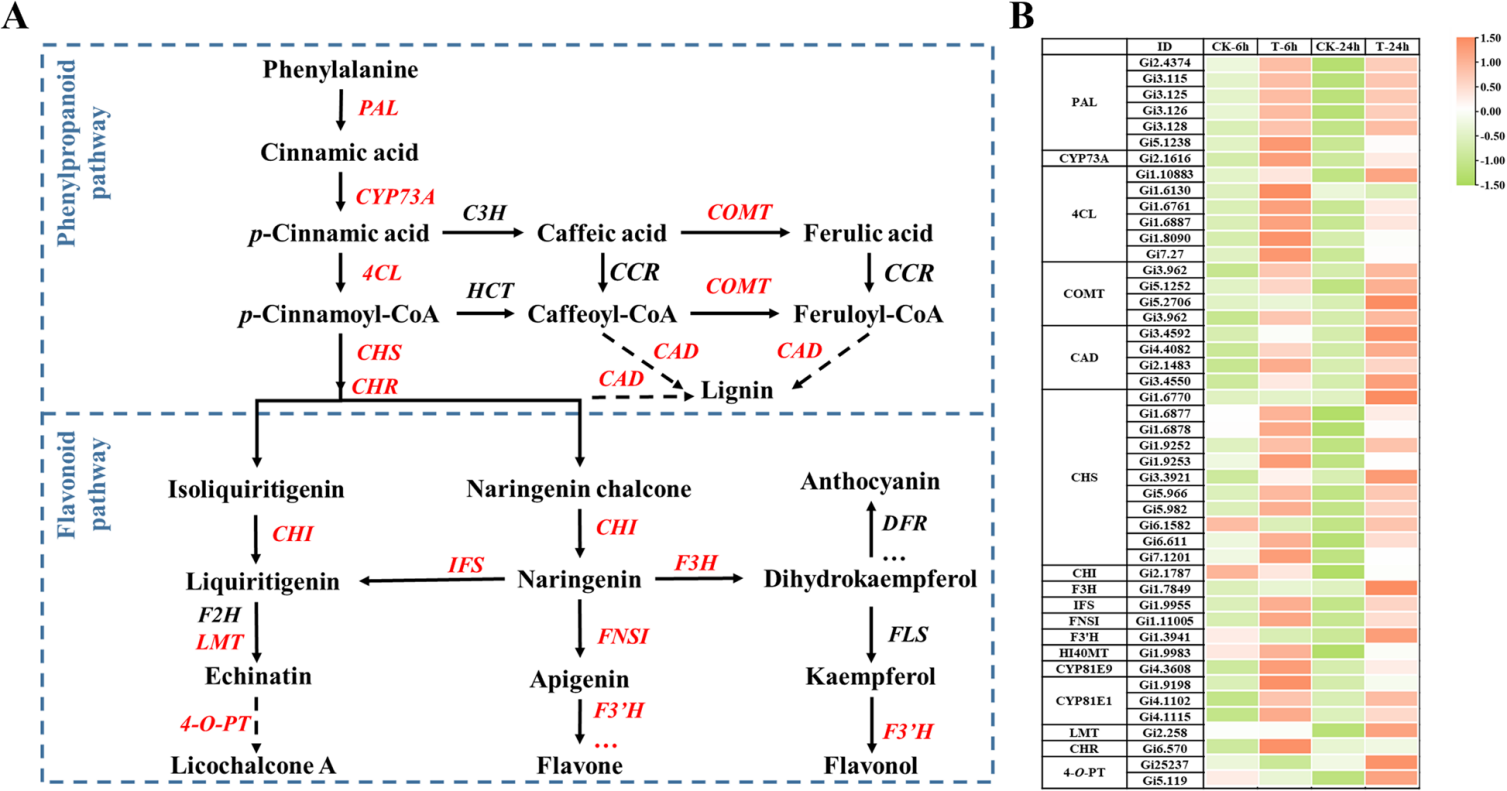
The DEGs involved in phenylalanine and flavonoid biosynthesis pathways under SAHA treatment. **A** Reconstruction of phenylalanine and flavonoid biosynthetic pathways with the DEGs. PAL, phenylalanine ammonia lyase. CYP73A, *trans*-cinnamate 4-monooxygenase. 4CL, 4-Coumarate-CoA ligase. COMT, caffeate *O*-methyltransferase. CAD, cinnamyl-alcohol dehydrogenase. CHS, chalcone synthase. CHI, chalcone isomerase. IFS, isoflavone synthase. F3H, flavanone-3-hydroxylase, F3’H, flavonoid 3’-hydroxylase. FNSI, Flavone synthetase type I. HI4OMT, 2,7,4’-trihydroxyisoflavanone 4’-*O*-methyltransferase. CYP81E9, isoflavone 3’-hydroxylase. CYP81E1, isoflavone 2’-hydroxylase. **B** Heatmap of the DEGs involved in phenylalanine and flavonoid biosynthesis. The heatmap colors range from green to red, representing the normalized log_2_ (FPKM) values of each gene

The study compared expression patterns of genes involved in phenylpropanoid and flavonoid biosynthesis in SAHA-treated and untreated seedlings. Figure 2 illustrates that 41 genes were up-regulated under SAHA treatment, with 20 genes specifically associated with phenylpropanoid biosynthesis and the remainder involved in flavonoid biosynthesis. Notably, *PAL* regulates the rate-limiting step in phenylpropanoid biosynthesis, while *CHS* performs a similar function in flavonoid biosynthesis. Additionally, several other key enzymes including *CYP73A*, *4CL*, *CHI*, *IFS*, *F3H*, *FNSI*, *F3'H*, *HI4OMT*, and *CYP81* were also up-regulated. These up-regulated genes likely contributed to the enhanced accumulation of flavonoids under SAHA treatment.

Transcription factors (TFs) from various families, including AP2/ERF, bHLH, bZIP, MYB, WRKY, NAC, and SPL, are known to regulate the biosynthesis of specialized metabolites in plants (Khan et al. 2023). This study identified 396 up-regulated and 225 down-regulated TFs, comprising members from the MYB, ERF, WRKY, bHLH, NAC, and bZIP families (Fig. S7). Notably, the number of up-regulated TFs significantly exceeded the number of down-regulated ones following SAHA treatment. These differentially expressed TFs may be responsible for the transcriptome alterations observed after SAHA treatment.

Phytohormones, including ABA, JA, and ethylene, play pivotal roles in regulating the biosynthesis of specialized metabolites in plants. To elucidate the underlying regulatory mechanisms, this study analyzed the expression profiles of 51 genes involved in plant hormone signal transduction in *G. inflata* seedlings treated with SAHA (Fig. S8 and Table S2). Notably, genes associated with ABA, JA, and ethylene pathways exhibited predominant up-regulation, suggesting their significance in SAHA-induced responses.

**Fig. 3:**
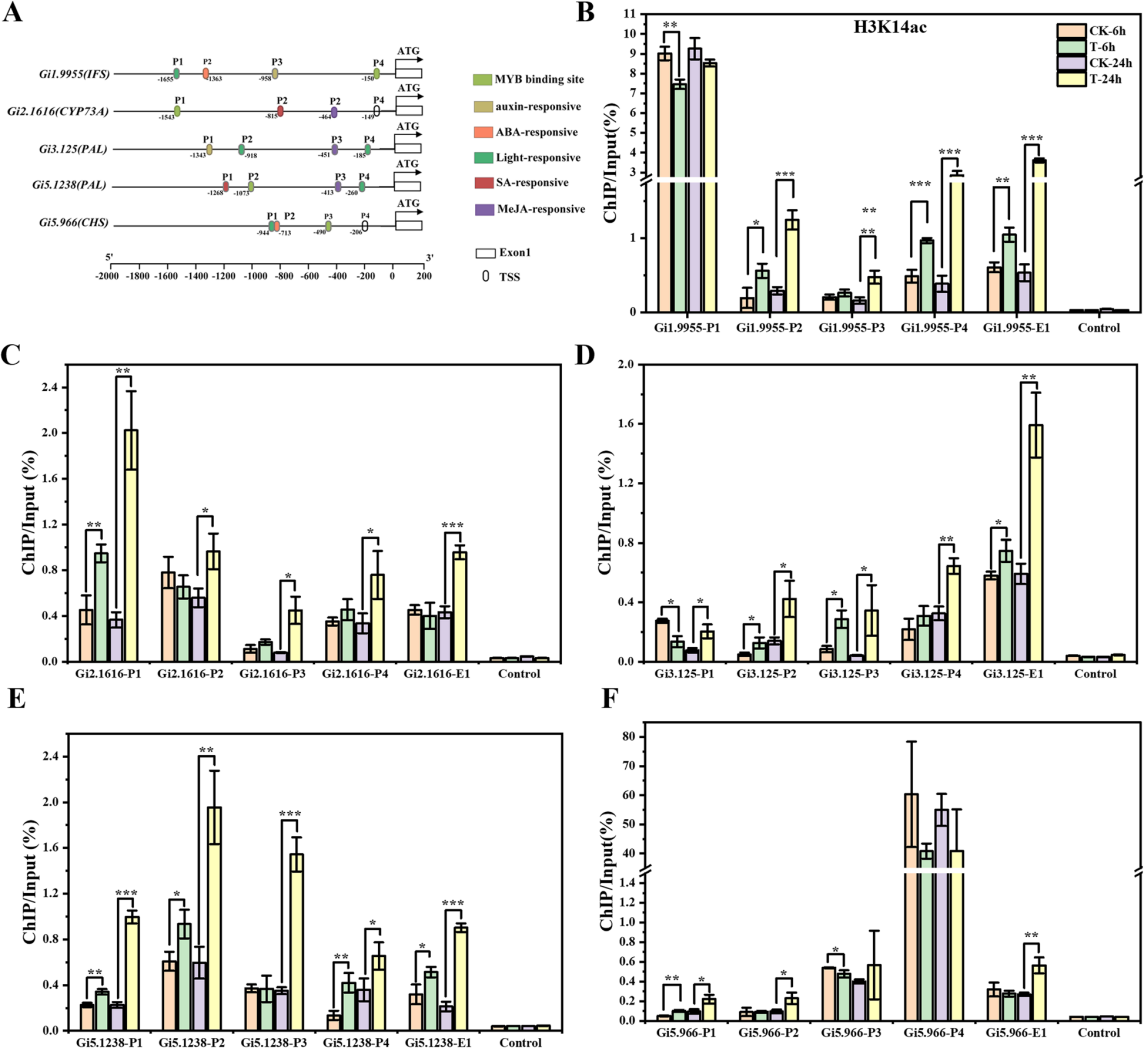
SAHA treatment enhanced H3K14ac levels at promoters of flavonoid biosynthesis genes. **A** Schematic diagram illustrating primer locations for ChIP-qPCR. H3K14ac level detection at *GiIFS* (*Gi1.9955*) (**B**), *GiCYP73A* (*Gi2.1616*) (**C**), *GiPAL* (*Gi3.125*) (**D**), *GiPAL* (*Gi5.1238*) (**E**) and *GiCHS* (*Gi5.966*) (**F**) loci in 7-day-old *G. inflata* seedlings treated with SAHA or DMSO (control) for 6 h and 24 h, as determined by ChIP‐qPCR assay. Values represent means ± SD. Student’s *t*-test, *P < *0.05*, ** P < 0.01, *** P < 0.001, n ≥ 3

To investigate the effect of SAHA treatment on the H3 acetylation levels of genes involved in flavonoid biosynthesis, we observed that SAHA treatment increased both the H3 acetylation levels (Fig. 1H) and the expression of flavonoid biosynthesis-related genes (Fig. 2). As histone acetylation primarily occurs on proximal promoters or exons (Ding et al. 2012), we conducted ChIP-qPCR analysis to examine the H3 acetylation levels at the promoter region of five representative genes (*GiIFS*, *GiCYP73A*, *GiPAL*, *GiPAL* and *GiCHS*). Primers were designed for specific fragments based on the analysis of common cis-acting elements in the promoters of these genes (Fig. 3A). A ChIP assay with a nonspecific rabbit IgG antibody served as a negative control. As illustrated in Fig. 3B-F and Fig. S9-11B-F, SAHA treatment significantly elevated the levels of H3ac (Fig. S9), H3K9ac (Fig. S10), H3K14ac (Fig. 3), and H3K27ac (Fig. S11) at the promoter regions of *GiIFS*, *GiCYP73A*, *GiPALs*, and *GiCHS*. This finding suggests that SAHA treatment may activate the expression of flavonoid biosynthesis-related genes, at least partially, by increasing the acetylation level of H3 at their promoters.

### HDACs in *G. inflata*

**Fig. 4:**
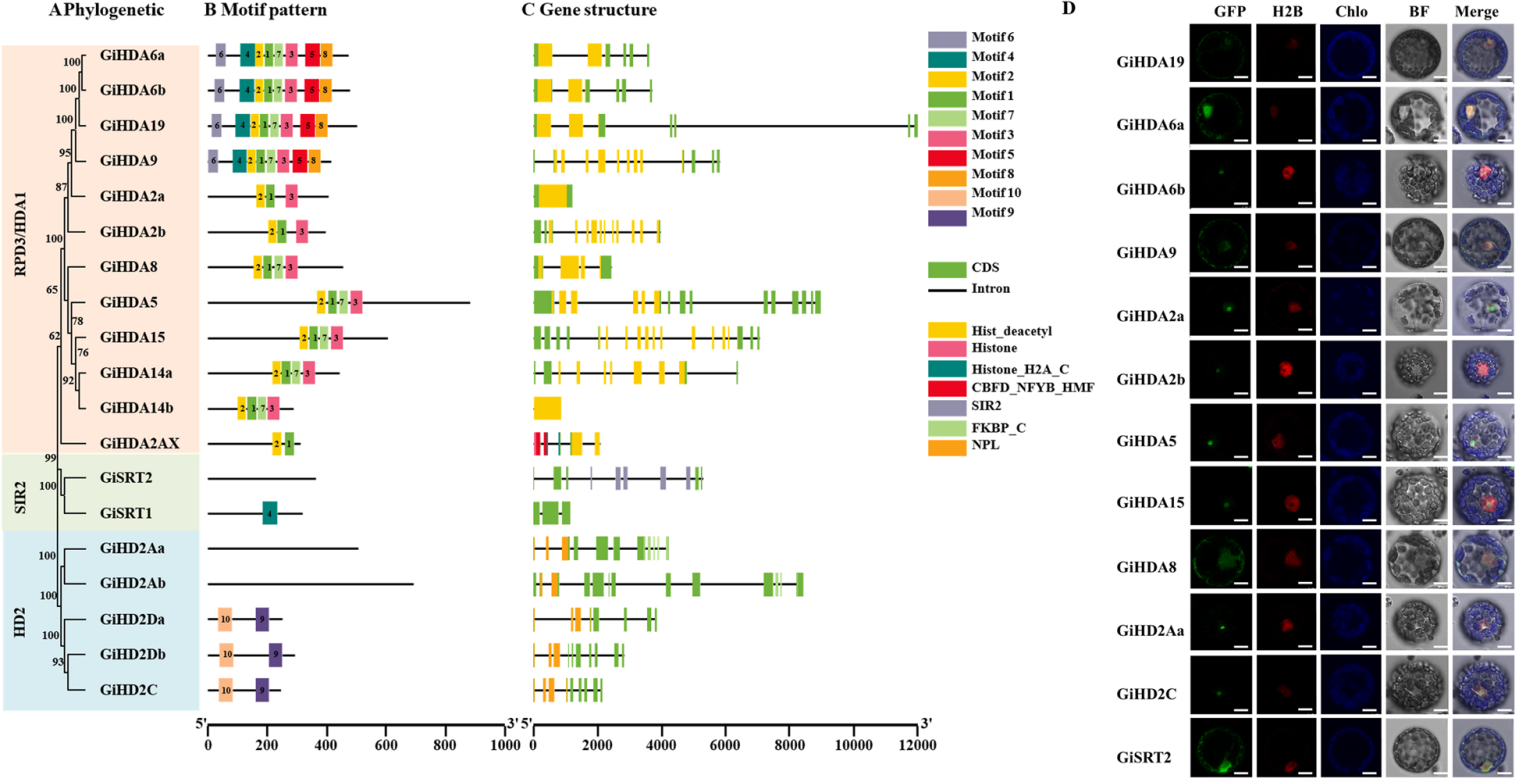
Characterization of *GiHDACs.* **A** Phylogenetic tree construction based on the full-length sequence of GiHDAC proteins. Colors denote subfamilies: red for RPD3/HDA1, blue for HD2, and green for SIR2. **B** Distribution of motifs in each GiHDAC protein. Motifs and their gene locations are labeled and classified according to subfamilies. **C** Gene structure and conserved domain distribution of GiHDACs. Green boxes represent unrelated areas, black lines indicate introns, yellow boxes show HDAC domains, orange boxes denote NPL domains, and purple boxes represent SIR2 domains. **D** Subcellular localization of GiHDACs. *GiHDACs*-GFP fusion constructs were utilized to determine the subcellular localization of GiHDACs. NLS-mCherry served as a nuclear marker. Scale bars = 10 μm

HDACs play crucial roles in various aspects of plant growth and responses to environmental stimuli. In this study, we identified 19 HDACs in the *G. inflata* genome. Based on their sequence similarity to homologs in *Arabidopsis* and other species, GiHDACs were classified into three families: RPD3/HDA1 family, SIR2 family, and HD2 family (Fig. 4A; Fig. S12). The RPD3/HDA1 family, homologous to yeast RP3 and HDA1, comprised 12 GiHDACs and represented the largest family. These members possess a typical HDAC domain that requires a zinc ion for catalytic activity (Hollender et al. 2008). The SIR2 family, which requires nicotine adenine dinucleotide (NAD^+^) as a co-factor (Gallo et al. 2004), was represented by only 2 SRT proteins in *G. inflata*. The HD2 family, initially identified in maize, was absent in yeast and animals (Fig. 4C)*.* In *G. inflata*, the HD2 family members (*GiHD2Aa*, *GiHD2Ab*, *GiHD2C*, *GiHD2Da*, and *GiHD2Db*) contained a conserved terminal amino acid region known as the EFWG motif. To further analyze the motifs presented in GiHDAC proteins, we conducted MEME analysis and constructed a phylogenetic tree using MEGA7. We identified a total of 10 motifs (Table S3). As shown in Fig. 4B, all 12 members of the RPD/HDA1 subfamily contained motifs 1, 2, and 3. The five members of the HD2 subfamily possessed motif 9 and motif 10, while GiSRT2 contained motif 4. The significant differences in conserved motifs of GiHDACs suggest diverse functions for these proteins.

Among the GiHDAC proteins, GiHDA5 was the largest, comprising 812 amino acids (aa), while GiHD2Aa was the smallest at 293 aa. The molecular masses of GiHDACs ranged from 31.76 kDa to 90.17 kDa, with isoelectric points (pI) varying from 4.65 (GiHD2Ab) to 10.09 (GiHDA2AX) (Table S4). Furthermore, the *GiHDAC* genes were mapped to their respective *G. inflata* chromosomes. The 19 *GiHDAC* genes were distributed across chromosomes 1, 3, 4, 6, 7, and 8, with chromosome 1 containing the highest number (Fig. S13). Analogous to *Arabidopsis *(Hollender et al. 2008), no discernible patterns in the chromosomal distribution of the three families were observed in the *G. inflata* genome.

As predicted by WoLF PSORT, GiHDAC proteins potentially localized in the cytosol, nucleus, chloroplast, and vacuole (http://www.genscript.com/psort/wolf_psort.html) (Table S4). Transient expression of GiHDAC-GFP fusion proteins in protoplasts of *Nicotiana benthamiana* was conducted. As illustrated in Fig. 4D, three members of the RPD3/HDA1 family, GiHDA9, 19, and 6a localized in both the cytosol and nucleus. Conversely, five RPD3/HDA1 family members (GiHDA5, 15, 2a, 2b, and 6b) primarily localized in the nucleus, specifically in the nucleolus, aligning with the predicted localization. Additionally, GiHDA8 and GiSRT2 were detected in both the cytosol and nucleus, with predominant nuclear localization (Fig. 4D). Furthermore, two members of the HD2 family, GiHD2Aa and GiHD2C, localized in the nucleus. The diverse localization patterns observed for GiHDACs suggest their distinct cellular functions.

### GiHDACs involved in flavonoid accumulation

Licorice thrives in cold, arid regions with saline-alkaline soil, which influences the accumulation of its distinctive specialized metabolites. Methyl jasmonic acid (MeJA) and ABA are plant hormones that regulate plant growth and stress responses, while HDACs have been identified as crucial regulators for these processes. To examine the effects of environmental stresses on specialized metabolite accumulation, licorice growth, and *GiHDAC* expression, we subjected *G. inflata* seedlings to various treatments. These included salt (250 mM NaCl), alkaline (NaHCO_3_, pH8.0), and cold (4 ℃) conditions, as well as plant hormones, specifically 100 μM MeJA and 100 μM ABA, to assess their impact on licorice growth and production.

**Fig. 5:**
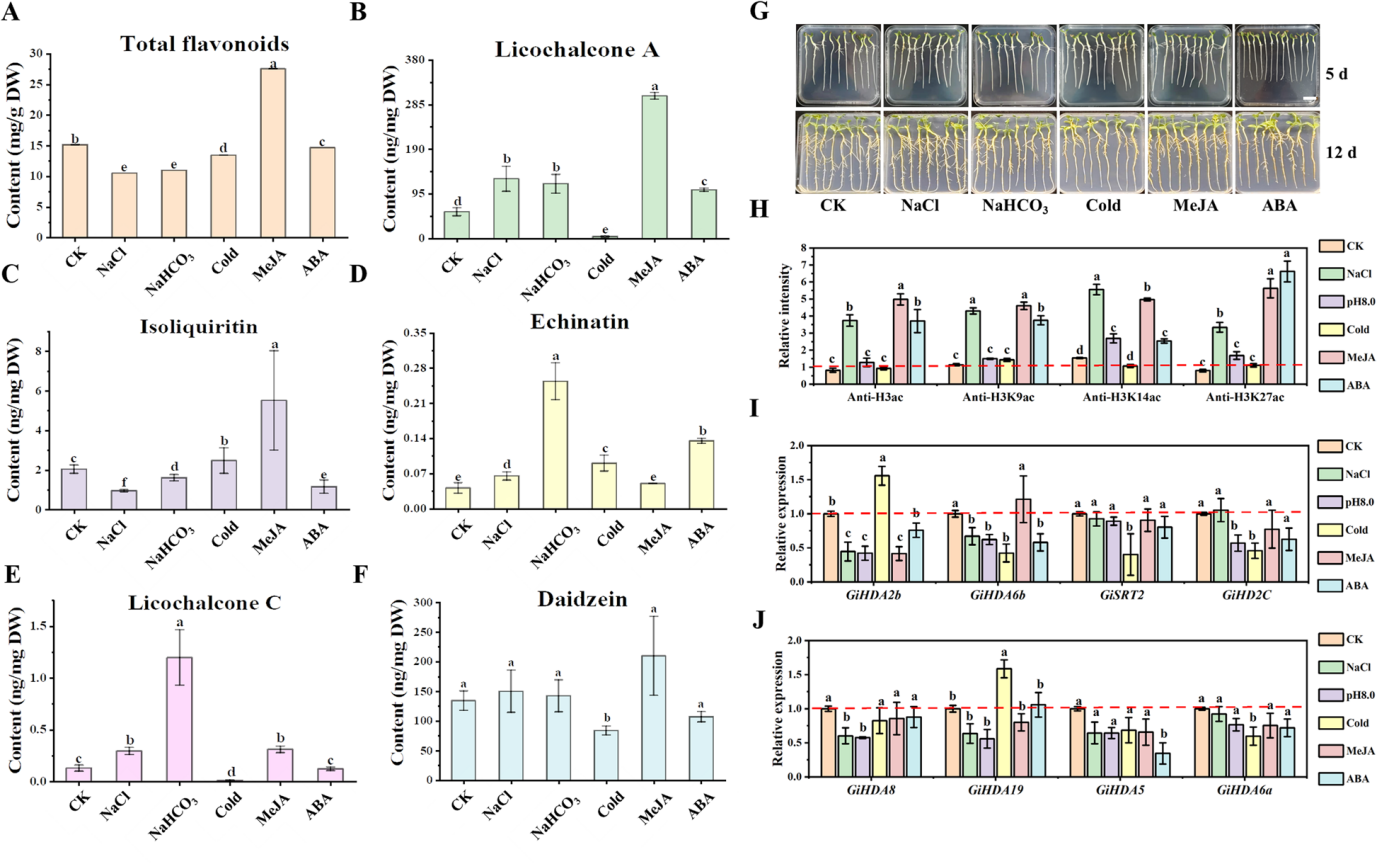
Expression profiles of *GiHDAC* genes and histone acetylation status under different treatments. The content of total flavonoids (**A**), LCA (**B**), isoliquiritin (**C**), echinatin (**D**), licochalcone C (LCC) (**E**), and daidzein (**F**) in *G. inflata* seedlings under various treatments were quantified using HPLC. (**G**) The morphology of *G. inflata* seedlings under different treatments. Five-day-old *G. inflata* seedlings were exposed to ethanol (Control), 250 mM NaCl, NaHCO_3_(pH 8.0), cold (4℃), 100 μM MeJA and 100 μM ABA for 7 days. Scale bar = 2.0 cm. **H** The relative intensity was calculated as the ratio of histone H3 acetylation to total histone H3 using ImageJ. **I, J** qRT-PCR analysis of gene expression. Different lower-case letters indicate statistically significant differences at a P-value of 0.05 for the relative expression level and the flavonoid contents among samples. Student's *t*-test, n ≥ 3. The y-axis depicts expression levels relative to the control, which was set to 1.0 (indicated with a dashed line). **I** and **J** include 8 representative *GiHDAC* genes from different families

To examine the impact of stress on the accumulation of specific metabolites in *G. inflata*, we quantified the levels of total flavonoids, LCA, isoliquiritin, echinatin, licochalcone C (LCC), and daidzein in the roots. Our results demonstrated that MeJA enhanced the levels of most compounds tested, while cold treatment decreased the accumulation of LCA, LCC, and daidzein (Fig. 5A-F). Additionally, we assessed histone acetylation levels under various treatments. Western blot analysis revealed that protein acetylation levels of H3, particularly H3K9, H3K14, and H3K27, increased in response to salt, MeJA, and ABA treatments (Fig. 5H and Fig. S14).

To further elucidate the response of *GiHDAC* genes to abiotic stress conditions, we conducted expression analysis using qRT-PCR for eight representative *GiHDAC* genes from different families. Figure 5G illustrates the plant phenotype after one week of exposure to salt, alkali, low temperature, MeJA, and ABA stress. Subsequent quantitative assessment of root elongation revealed that both cold stress and ABA treatment significantly inhibited root growth, resulting in a fresh weight approximately 60% of that observed in the control group (Fig. S15). The *GiHDAC* genes demonstrated active responses to the treatments. Under salt stress, four *GiHDAC* genes (*GiHDA2b*, *GiHDA8*, *GiHDA19*, and *GiHDA15*) exhibited down-regulation, while the expression of *GiHDAC* genes (*GiHDA6b*, *GiSRT2*, *GiHD2C*, and *GiHDA6a*) remained constant. In response to alkaline stress, six examined *GiHDACs* showed decreased expression levels (except *GiSRT2* and *GiHDA6a*), with *GiHDA2b* being the most affected. Additionally, four *GiHDAC* genes (*GiHDA6b*, *GiSRT2*, *GiHD2C*, and *GiHDA6a*) were strongly repressed under cold stress, while the expressions of *GiHDA2b* and *GiHDA19* were significantly induced. However, *GiHDA8* and *GiHDA5* did not exhibit significant changes in expression under low temperature. Regarding plant hormone treatments, most of the 19 *GiHDAC* genes showed down-regulation in expression levels after 2 and 6 h of MeJA treatment (Fig. S16B). However, after 24 h of MeJA treatment, only *GiHDA2b* exhibited decreased expression, while the expression levels of other *GiHDACs* were comparable to the control (Fig. 5I, J). Under ABA treatment, the expression levels of *GiHDA6b* and *GiHDA5* were down-regulated.

Furthermore, we investigated the spatio-temporal expression patterns of *GiHDACs* to elucidate their potential influence on the physiological functions of *G. inflata* growth and development. As illustrated in Fig. S16A, the majority of *GiHDACs* demonstrated higher expression levels in roots compared to leaves, with their expression levels increasing over time. Notably, *GiHDA14a*, *GiHDA5*, and *GiSRT2* exhibited elevated transcript accumulation in leaves, while expression in other organs remained low. This suggests the involvement of *HDACs* in regulating metabolic biosynthesis in *G. inflata*.

### *GiHDA2b* negatively regulated flavonoid accumulation and H3 acetylation levels

**Fig. 6:**
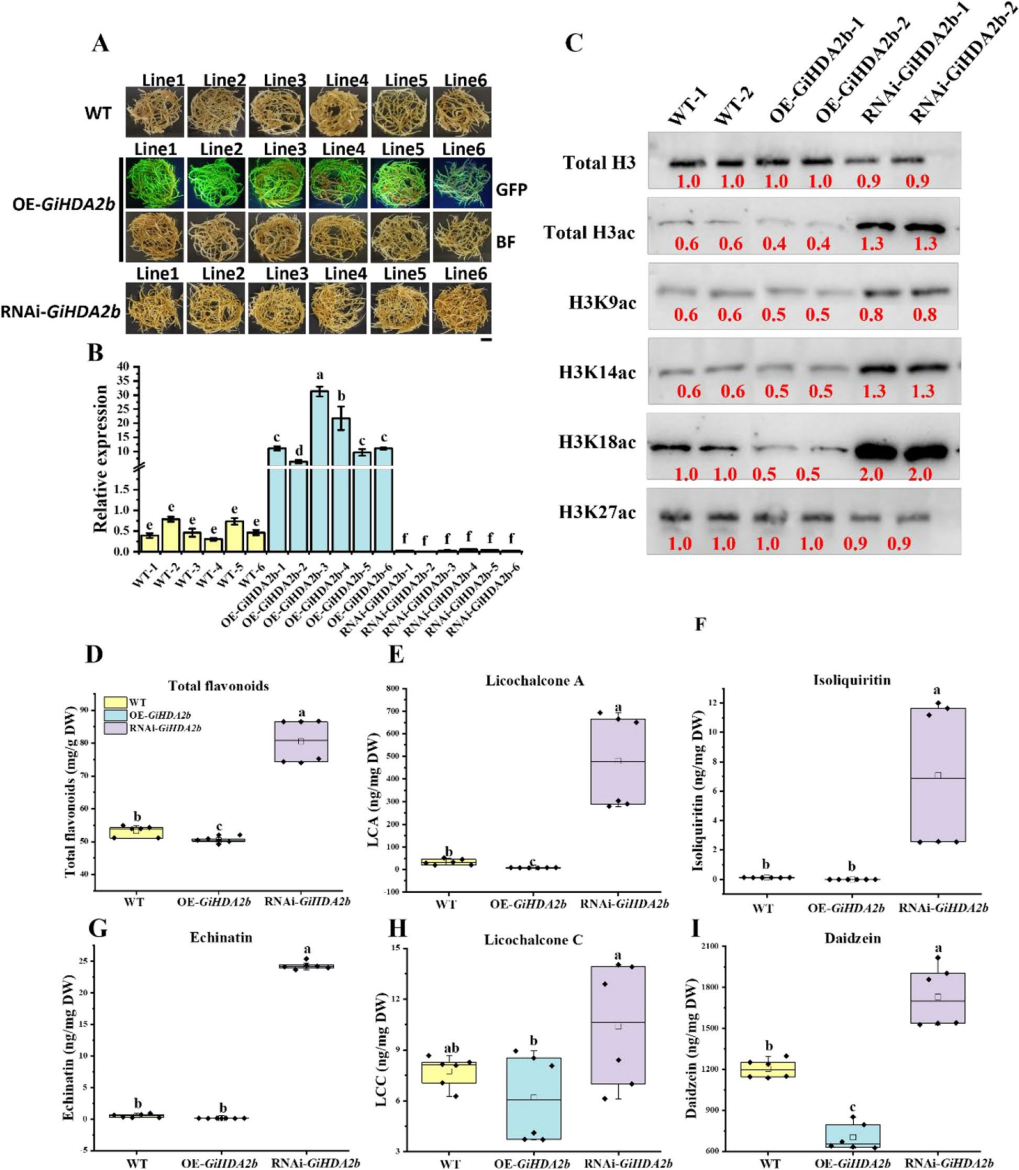
Analysis of flavonoids contents in OE-*GiHDA2b* and RNAi-*GiHDA2b* transgenic hairy roots. **A** The phenotype of the OE-*GiHDA2b* and RNAi-*GiHDA2b* transgenic hairy roots (scale bars: 1 cm). **B** Expression level of *GiHDA2b* in the OE-*GiHDA2b* and RNAi-*GiHDA2b* lines as determined by qRT-PCR. **C** Immunoblot analysis of histone acetylation in WT, OE*-GiHDA2b* and RNAi-*GiHDA2b* hairy roots. ImageJ software was utilized to quantify band intensities. Histone acetylation levels were detected using antibodies against H3ac, H3K9ac, H3K14ac, H3K18ac, and H3K27ac, with histone H3 serving as the internal control for nuclear protein loading. The red numbers indicate the relative quantitative values of band intensities. The content of total flavonoids (**D**), LCA , isoliquiritin (**F**), echinatin (**G**), licochalcone C (**H**) and daidzein (**I**) in OE-*GiHDA2b* and RNAi-*GiHDA2b* transgenic hairy roots were quantified by HPLC. The non-transgenic hairy roots were designated as WT. The different lower-case letters indicate a significant difference at the 0.05 level for the relative expression level and flavonoids content among samples. Student’s *t*-test, *n* = 3

MeJA (Fig. 5) and SAHA (Fig. 1) treatments demonstrated the most significant induction of flavonoid contents. Although HDACs exert their effects through various mechanisms, the molecular mechanisms by which MeJA promotes the accumulation of flavonoids in licorice during histone deacetylation have not been sufficiently investigated. In this study, the expressions of all *GiHDACs* were suppressed at 2 h and 6 h post-MeJA treatment; however, after 24 h, only the expression of *GiHDA2b* was inhibited by MeJA (Fig. S16B and 5I), suggesting that *GiHDA2b* may be one of the primary targets through which MeJA influences flavonoid synthesis in licorice. Consequently, we proceeded to clone this gene and analyze its function. Both OE-*GiHDA2b* and RNAi-*GiHDA2b* transgenic hairy roots of *G. inflata* were generated and confirmed using PCR (Fig. S17A), western blot (Fig. S17B), GFP fluorescence (Fig. 6A) and qRT-PCR assays (Fig. 6B).

To confirm the hypothesized histone deacetylase function of GiHDA2b, western blot analysis was conducted to examine the acetylation levels of H3 in wild-type (WT), OE-*GiHDA2b*, and RNAi-*GiHDA2b* lines. The results revealed that the acetylation levels of H3K9ac, H3K14ac, and H3K18ac were significantly decreased in OE-*GiHDA2b* compared to WT, while they were notably increased in RNAi-*GiHDA2b* relative to WT (Fig. 6C; Fig. S18). Collectively, these findings suggest that GiHDA2b may function as an active histone deacetylase in *G. inflata.*

In comparison to the WT and OE-*GiHDA2b* lines, the RNAi-*GiHDA2b* hairy roots displayed a more pronounced yellow coloration, which aligned with the elevated levels of total flavonoids observed in the RNAi-*GiHDA2b* lines (Fig. 6A, D). This observation suggests a negative regulatory role of GiHDA2b in flavonoid accumulation. Additionally, the RNAi-*GiHDA2b* transgenic hairy roots exhibited higher concentrations of LCA, isoliquiritin, echinatin, LCC, and daidzein when compared to the WT control and OE-*GiHDA2b* lines (Fig. 6E-I). An examination of one-month-old *GiHDA2b* transgenic hairy roots revealed no significant variations in fresh weight among the WT, OE, and RNAi lines (Fig. S19). These results collectively indicate that GiHDA2b negatively regulates the accumulation of LCA and total flavonoids in *G. inflata* hairy roots.

### GiHDA2b regulated LCA biosynthesis by modulating genes marked with H3K18ac

Acetylation modification plays a crucial role in regulating gene expression in plants. Among the tested N-terminal lysine residues of histone H3, the acetylation of H3K18 was significantly inhibited or enhanced in the OE and RNAi of *GiHDA2b* (Fig. 6C). H3K18ac is associated with the transcription enhancer, primarily located in the region surrounding the transcription start site (Wang et al. 2008). Furthermore, studies have indicated that HDACs may negatively regulate flavonoid biosynthesis by modulating the acetylation levels of histone H3 (Bai et al. 2016; Sun et al. 2023). This suggests that GiHDA2b may suppress the transcription of LCA biosynthesis-related genes by removing the H3K18 acetylation modification on the chromatin of gene promoters, thereby exerting a negative regulatory effect on LCA biosynthesis. To validate this hypothesis, ChIP-qPCR was employed to assess the levels of H3K18 acetylation on the promoters of genes associated with the LCA biosynthesis pathway, including 8 structural genes: *GiPAL* (*Gi3.125*), *GiCHS* (*Gi5.966*), *GiCHS4* (*Gi6.1582*), *GiCHR* (*Gi6.570*), *GiCHI* (*Gi2.1787*), *GiF2H2* (*Gi1.11009*), *GiLMT* (*Gi2.258*), and *GiPT* (*Gi7.1190*). Primers were designed to target specific segments based on the analysis of common cis-acting elements within their promoters (Fig. S20). As illustrated in Figure S21, the acetylation levels of histones at the promoters of these 8 structural genes were highest in the RNAi lines compared to WT and OE. qRT-PCR results revealed that in OE-*GiHDA2b*, the expression levels of these genes were significantly down-regulated, whereas in RNAi-*GiHDA2b*, they were markedly up-regulated (Fig. S22). These findings suggest that the enhanced LCA content in the RNAi transgenic hairy roots may be attributed to the reduced enzymatic activity of GiHDA2b, leading to an elevation in H3K18 acetylation modification levels and subsequently promoting the expression of genes involved in the LCA biosynthesis pathway.

## Discussion

Licorice flavonoids are typically present in small quantities, making their isolation challenging. For instance, only 8 mg of LCA can be extracted from 0.5 kg of dried roots of *G. inflata *(Yang et al. 1990). Furthermore, artificial synthesis methods involve complex process routes. LCA production requires a four-step reaction, including methylation, aldol condensation, isomerization, and alkaline hydrolysis of esters, resulting in a mere 4.1% total yield (Wang et al. 2004). The high production costs of these natural products hinder the industrial-scale production of individual flavonoids. This limitation subsequently impedes the development of LCA and comprehensive pharmaceutical research. Consequently, enhancing LCA biosynthesis and accumulation through biotechnological methods assumes greater importance.

Previous research indicates that HDACs regulate plant growth and response to environmental stress by modulating acetylation levels of histones and other proteins (Ma et al. 2013). In this study, we observed that HDAC inhibitors enhanced flavonoid accumulation in *G. inflata*, a medicinal licorice (Fig. 1A-G), potentially by increasing histone H3 acetylation levels in the promoters of genes related to flavonoid biosynthesis (Fig. 1H; Fig. 2). Notably, flavonoid biosynthesis was typically affected by salt, alkali, and cold stress in natural conditions, with concomitant changes in H3 acetylation levels during these treatments.

**Fig. 7:**
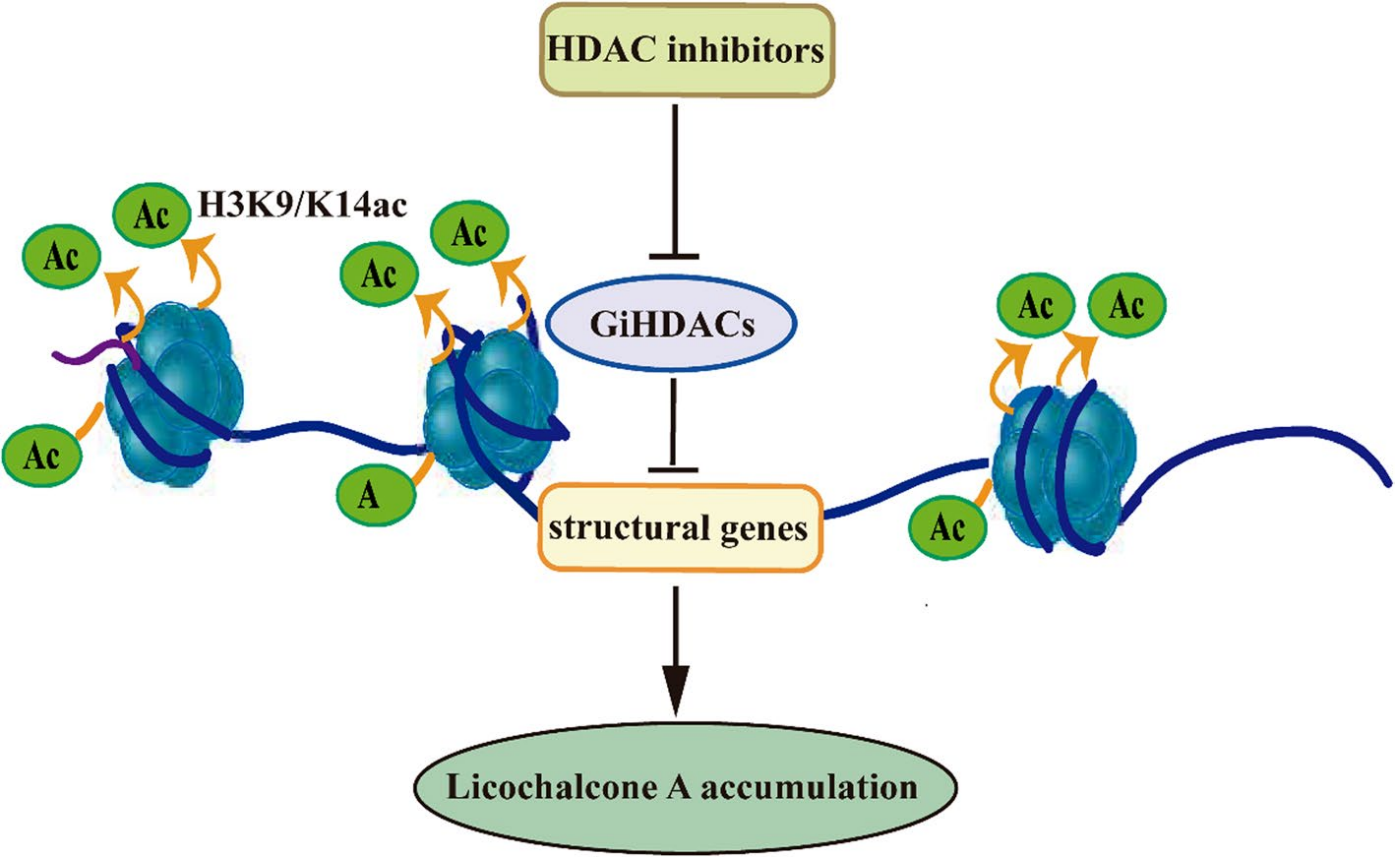
Proposed model for GiHDACs regulating LCA accumulation. GiHDACs reduce the acetylation level of histone H3 at lysine residues in LCA biosynthetic genes, thereby inhibiting the expression of these genes and reducing the accumulation of LCA. HDAC inhibitors, on the other hand, counteract the inhibitory effect of GiHDACs on LCA accumulation by inhibiting the deacetylase activity of GiHDACs

This study demonstrated that SAHA treatment enhanced flavonoid biosynthesis and elevated H3 acetylation levels in *G. inflata* seedlings. RNA-seq analysis identified 2,781 DEGs (Fig. S4), with notable up-regulation of genes involved in flavonoid biosynthesis (Fig. 2). Subsequent ChIP-qPCR analysis revealed increased H3 acetylation levels in the promoter regions of five genes associated with flavonoid biosynthesis under SAHA treatment (Fig. 3; Fig. S9-11). Moreover, SAHA treatment activated a group of transcription factors (Fig. S7). The up-regulation of key enzymes and transcription factors involved in flavonoid biosynthesis suggests that SAHA treatment enhances flavonoid accumulation by increasing histone acetylation levels, thereby activating the expression of these genes (Fig. 7). SAHA is a pioneering HDAC inhibitor, while TSA primarily targets class I and II HDACs. NaB predominantly acts on class I and II HDACs, whereas SIR and NIC primarily affect class III HDACs (Patanun et al. 2016). Considering that SAHA, NaB (Fig.1), and NIC (Zeng et al. 2023) treatments all increased flavonoid contents in *G. inflata* seedlings, multiple HDACs may be involved in regulating flavonoid biosynthesis.

Epigenetic regulation of plant specialized metabolism draws more and more attention (Sun et al. 2022) and the advancement of bioinformatic techniques greatly facilitates research in non-model plants (Zheng et al. 2023). Recent decades have witnessed increased attention to the identification and characterization of HDAC genes across various plant species (Pandey et al. 2002). In this study, we identified 19 GiHDACs in the *G. inflata* genome and categorized them into three families based on sequence similarity to homologs in other species: the RPD3/HDA1 family, the SIR2 family, and the HD2 family, each exhibiting distinct features and functional domains (Fig. 4 and Fig. S12). The distribution of HDAC genes on *G. inflata* chromosomes appeared random (Fig. S13). Moreover, we identified conserved motifs in the HDAC families, providing insights for a more comprehensive understanding of their functions and evolutionary relationships. Subcellular localization analysis revealed that GiHDACs could be found in various cellular compartments, suggesting their involvement in diverse cellular processes (Fig. 4D). HDACs have been implicated in responses to various abiotic stresses including cold, salt, and drought. For instance, *AtHDA9* regulates the histone acetylation levels of numerous stress-responsive genes to negatively modulate plant sensitivity to salt and drought stress (Zheng et al. 2016). In *Arabidopsis*, the *AtHDA6* mutant *axe1-5* and *At* *HDA6*RNA interference plants demonstrate increased sensitivity to ABA and salt stress (Chen et al. 2010). Similarly, overexpression of *OsHDT701* enhances tolerance to drought and salt stress in rice (Cho et al. 2018). In this study, we observed alterations in the accumulation of specific metabolic products in response to stress (Fig.5A-F). Furthermore, we identified changes in the acetylation levels of histones, and noted that the *HDAC* genes in *G. inflata* exhibited diverse responses to various stress conditions (Fig. 5I, J), indicating their potential involvement in stress tolerance mechanisms.

Plant hormones serve as crucial regulators for plant responses to biotic and abiotic stresses, as well as for specialized metabolism. Notably, SAHA treatment up-regulated the expression of genes involved in plant hormone signaling pathways, particularly those related to ABA, JA, and ethylene (Fig. S8). In line with these findings, both MeJA and ABA treatments enhanced the accumulation of flavonoids in *G. inflata* seedlings (Fig. 5A-F). Western blot analysis revealed that the acetylation levels of histone H3, specifically at H3K9, H3K14, and H3K27, increased under salt, MeJA, and ABA treatments (Fig. 5H and Fig. S14). The expression of GiHDACs was inhibited by MeJA and ABA treatments. These results suggest that plant hormones such as MeJA or ABA may modulate histone acetylation levels by suppressing the expression of HDACs and activating flavonoid biosynthesis-related genes, thereby promoting the accumulation of flavonoids in response to environmental changes.

In our previous research, we discovered that the HDAC inhibitor NIC enhanced flavonoid biosynthesis by inhibiting GiSRT2 activity. The present study revealed that the expression of *GiHDA2b* was stimulated by SAHA and MeJA treatments and played an inhibitory role in regulating flavonoid biosynthesis in *G. inflata.* Overexpression of *GiHDA2b* decreased flavonoid accumulation, whereas RNAi-mediated suppression of *GiHDA2b* increased flavonoid levels (Fig. 6D-I). Additionally, we observed that *GiHDA2b* was localized in the nucleus (Fig. 4D) and functioned as a potential active histone deacetylase (Fig. 6C). Moreover, the results of ChIP-qPCR (Fig. S21) and qRT-PCR (Fig. S22) indicated that GiHDA2b regulated the expression levels of LCA biosynthesis-related genes by inhibiting the H3K18 acetylation modification on histones near the promoters of these genes. These findings elucidate the transcriptional regulatory mechanism of specific genes by GiHDA2b in *G. inflata*, providing valuable insights for further research on the regulation of plant secondary metabolic pathways. Given that HDAC inhibitors NIC, SAHA, and NaB all enhanced flavonoid biosynthesis, it is plausible to hypothesize that additional GiHDACs may act as negative regulators of this process. It is also conceivable that some GiHDACs may redirect the flavonoid biosynthesis pathway to branches not examined in this study, and GiHDACs may promote the accumulation of specific flavonoid compounds. However, understanding the mechanisms through which HDAC inhibitors enhance total flavonoid content, in contrast to the suppressive effect of *GiSRT2* or *GiHDA2b* overexpression on total flavonoid accumulation, remains an intriguing biological question for future investigation.

Plants exhibit a remarkable capacity to detect environmental stimuli and modulate the accumulation of secondary metabolites (SMs) as an adaptive strategy for survival in changing conditions (Franzoni et al. 2023; Ren et al. 2023). The quality and efficacy of medicinal materials are predominantly determined by SM accumulation levels. Histone acetylation plays a crucial role in various transcriptional regulatory processes, including secondary metabolism. Recent studies have demonstrated that histone deacetylation is involved in regulating secondary metabolism across numerous plant species (Table S6) (Ahn et al. 2023; Patrick et al. 2021; Song et al. 2019; Zhao et al. 2022; Chen et al. 2024). These findings highlight the significance of investigating the epigenetic regulatory mechanisms governing plant secondary metabolism, particularly through molecular and chemical epigenetic modifications, as two key research directions.

Molecular epigenetic modifications are primarily achieved through genetic engineering techniques, such as the knockout or overexpression of genes associated with epigenetic regulation. In contrast, chemical epigenetic modifications involve the exogenous application of chemical modifiers, particularly HDAC inhibitors. These inhibitors can activate dormant biosynthetic genes, thereby enhancing the chemical diversity of plant SMs and potentially improving the quality and efficacy of medicinal materials. Consequently, a comprehensive investigation of the regulatory role of HDACs in the accumulation of SMs in plants holds significant theoretical and practical implications for enhancing the quality of medicinal materials and developing novel pharmaceuticals.

In conclusion, this study offers significant insights into the crucial role of histone acetylation in the specialized metabolism of *G. inflata*. Further research is necessary to elucidate the underlying molecular mechanisms of these processes.

## Materials and methods

### Plant materials and growth conditions

Licorice plants (*G. inflata*) aged one to three years were cultivated in fields at the Northwest Biologic Agriculture Center (Ningxia, China). The *G. inflata* seeds, supplied by Gansu Jinyoukang Pharmaceutical Technology Co., Ltd., underwent surface sterilization and cultivation as previously outlined (Wu et al. 2022). Transgenic hairy roots were induced on 7-day-old seedlings following established protocols (Zeng et al. 2023), and subsequently subjected to various treatments.

To evaluate the impact of various treatments on flavonoid accumulation, 7-day-old *G. inflata* seedlings were transferred to MS media with or without the specified treatment chemical substances. Following 7 days of treatment, roots were harvested for total flavonoid content analysis. Each experiment was conducted in triplicate with 3–5 replicates. We sourced SAHA (Beyotime: SC0231), TSA (Selleck: S1045), NaB (Beyotime: S1539), and Sirtinol (Yeasen: 53719ES25) for our study.

### Vector construction and subcellular localization analysis of GiHDACs

The coding sequences of *GiHDACs* were isolated from *G. inflata* cDNA and subsequently inserted into *pCambia1300-GFP* to create overexpression vectors. The resulting constructs were verified through sequencing (Qingke, China). For the generation of RNAi-*GiHDA2b* transgenic hairy roots, the *pRNAiGG* vector (Yan et al. 2012) was employed. A comprehensive list of all primers utilized in this study is provided in Table S6.

The subcellular localization of GiHDACs was verified through transient expression in protoplasts of *Nicotiana benthamiana*, following previously established protocols (Wu et al. 2009). An empty vector, *pCambia1300-GFP*, served as the control. The nuclear localization marker NLS-mCherry was co-transfected into the protoplasts. Following a 12–16 h incubation period, the localization of the target protein was examined using confocal fluorescence microscopy (Leica TCS SP8 STED 3X).

### Protein extraction and western blot

*G. inflata* seedlings and transgenic hairy roots were ground into fine powder in liquid nitrogen and mixed with extraction buffer (10% glycerol, 60 mM Tris pH 6.8, 2.5% beta-mercaptoethanol, 2% SDS, and 0.025% bromphenol blue), then denatured at 95 °C for 10 min. The supernatant containing total protein was obtained by centrifugation at 13,000 rpm for 10 min.

SDS-PAGE was employed to separate proteins, and nylon membranes were utilized for western blot analysis. The study used several antibodies, including anti-H3 (Millipore: 06–755), anti-acetylated H3 (Millipore: 06–599), anti-acetylated H3K9 (Millipore: 07–352), anti-acetylated H3K14 (Millipore: 07–353), anti-acetylated H3K18 (Millipore: 07–354), anti-acetylated H3K27 (Millipore: 07–360), and anti-GFP (Abcam: ab290). Immunoblotting with anti-H3 and H4 antibodies served as loading controls. The secondary antibody was anti-rabbit IgG conjugated with horseradish peroxidase (Abcam: ab6721). Western Lightning Plus ECL Chemiluminescence Substrate (Perkin Elmer: NEL103001EA) was used to detect the signal. The experiments were conducted in triplicate, and ImageJ software was utilized to quantify relative protein levels.

### Compound extraction and HPLC analysis

As detailed by Zeng et al. (Zeng et al. 2023), compound extraction was performed using ultrasound-assisted extraction, followed by analysis utilizing a reversed-phase C18 column (150 mm × 4.6 mm, 5 μm, Shimadzu, Kyoto, Japan) and an HPLC (LC-2030C, Shimadzu, Kyoto, Japan) system. The quantification of total flavonoid content employed the sodium nitrite-aluminum nitrate colorimetric method, with rutin serving as the standard (Hao et al. 2018).

### qRT-PCR and ChIP-qPCR

RNA extraction and quantitative reverse transcription PCR (qRT-PCR) were performed as previously described (Li et al. 2022). *GiCOPS3* and *GiDREB* served as internal reference genes (Li et al. 2021), with relative expression levels calculated using the 2^−ΔΔCT^ method. The chromatin immunoprecipitation (ChIP) experiment was conducted according to Hu et al. (Hu et al. 2020). In brief, 3 g of *G. inflata* seedling roots underwent cross-linking with 1% (v/v) formaldehyde. Subsequently, chromatin was sonicated into fragments of 200 to 500 bp and immunoprecipitated using antibodies against H3ac, H3K9ac, H3K14ac, or H3K27ac. ChIP IT® Protein G Magnetic Beads (Active Motif, 53,033) were utilized to harvest the immunocomplexes. Following extensive washing, the immunoprecipitated chromatin was de-cross-linked to release DNA for qPCR. A ChIP assay employing a nonspecific rabbit IgG antibody served as a negative control. Enrichment was expressed as the percent input for all tested genes. Table S5 lists all primers used in this study.

### Generation of transgenic hairy roots of *G. inflata*

Transgenic hairy roots were generated following a previously described protocol with slight modifications (Yan et al. 2012). The binary vectors *pCambia1300-GiHDA2b-GFP* and *RNAi-GiHDA2b* were introduced into the modified *Agrobacterium tumefaciens* strain MSU440. Non-transgenic hairy roots (WT) served as the control. Positive transgenic lines were identified through PCR using *rolB* (from Ri plasmid of MSU440) and gene-specific primers. All primers utilized are listed in Table S5.

### RNA sequencing and data analysis

Seven-day-old *G. inflata* seedlings underwent treatment with or without 100 μM SAHA for 6 and 24 h, followed by total RNA extraction using the Total RNA Extraction Kit (Invitrogen, Carlsbad, CA, USA). Gene Denovo Biotechnology Co., Ltd. (Guangzhou, China) conducted the library construction, sequencing, and initial analysis. Genes or transcripts with a false discovery rate (FDR) ≤ 0.05 and absolute fold change ≥ 2 were classified as DEGs. The study identified significantly enriched GO terms and KEGG pathways using a threshold of FDR (adjusted *P*-value) ≤ 0.05.

## Supplementary Information

Supplementary Material 1. Figure S1. (A) The morphology of *G. inflata* seedlings exposed to HDAC inhibitors. From left to right: 7-day-old *G. inflata* seedlings were subjected to DMSO (control), 1 mM NaB, 1 μM TSA, 10 μM SIR, and 100 μM SAHA treatments for 7 days, respectively. Scale bar = 2.0 cm. (B) The fresh weight of *G. inflata* seedling roots under various treatments. Different lowercase letters denote a significant difference in acetylation modification levels at *P* < 0.05. Student's *t*-test, n ≥ 3. Figure S2. Full scan (uncropped) western blots from supplementary figures. This figure presents uncropped full scans of western blots corresponding to the cropped versions displayed within the supplementary figures. Figure S3. Histone acetylation of *G. inflata* seedlings increased under SAHA treatment. The relative intensity was determined by the ratio of histone H3 acetylation to total histone H3 using ImageJ. Different lowercase letters indicate significant differences in acetylation modification levels at *P* < 0.05 among samples. Student's *t*-test, n ≥ 3. Figure S4. Differentially expressed genes identified from RNA-seq. (A) Principal component analysis clustering of RNA-seq samples. (B) Venn diagrams illustrating the quantity and overlap of DEGs (n = 3972) in CK-6 h_VS_T-6 h and CK-24 h_VS_T-24 h comparison groups. (C and D) Volcano plots depicting the number of up-regulated and down-regulated DEGs in CK-6 h_VS_T-6 h (C) and CK-24 h_VS_T-24 h (D). DEGs were defined by Log_2_ (Fold Change) ≥ 1, *P*-value ≤ 0.05. Each point represents a DEG. Red points indicate up-regulated DEGs, green points indicate down-regulated DEGs, and blue points represent genes with no significant difference. (E) Summary of significantly up- and down-regulated genes in both comparison groups. Figure S5. qRT-PCR validation of expression patterns for 8 DEGs identified from RNA-seq. (A) The expression patterns of 8 genes determined by qRT-PCR. *GiCOPS3* was used as the internal control. Values represent means ± SD. Student's *t*-test,*P < 0.05, ** P < 0.01, *** P < 0.001, n = 3. (B) Correlation analysis between RNA-Seq and qRT-PCR results (R^2^= 0.90, *P*-value < 0.01). Figure S6. Functional enrichment analysis of DEGs identified from RNA-seq. (A) Top 20 KEGG pathways enriched in up-regulated DEGs (2665). Numbers on the right of each column indicate the percentage of DEGs in the corresponding term relative to total up-regulated DEGs, with the *q*-value shown in brackets. (B) GO enrichment analysis of up-regulated DEGs across three ontology classes: molecular function, cellular component, and biological process. Figure S7. Transcription factors (TFs) that were differentially expressed under SAHA treatment. (A) Quantity of differentially expressed TFs. (B) The differential expression patterns of genes in MYB, NAC, and WRKY families. The expression levels of DEGs were normalized by log_2_ (FPKM). Figure S8. Expression profiles of genes involved in the plant hormone signal transduction pathway under SAHA treatment. The heatmap illustrates log_2_ (FPKM) values for each of the DEGs. Figure S9. SAHA treatment increases the levels of H3ac at the promoter of genes involved in flavonoid biosynthesis. (A) Schematic diagram illustrates the location of primers utilized for ChIP-qPCR. Detection of H3K14ac levels at *GiIFS* (*Gi1.9955*) (B), *GiCYP73A* (*Gi2.1616*) (C), *GiPAL* (*Gi3.125*) (D), *GiPAL* (*Gi5.1238*) (E) and *GiCHS* (*Gi5.966*) (F) loci in 7-day-old *G. inflata* seedlings treated with SAHA or DMSO (control) for 6 hours and 24 hours by ChIP‐qPCR assay. Values represent means ± SD. Student's *t*-test,*P < 0.05, ** P < 0.01, *** P < 0.001, n = 3. Figure S10. SAHA treatment increased the levels of H3K9ac at the promoter of genes involved in flavonoid biosynthesis. (A) Schematic diagram illustrates the location of primers utilized for ChIP-qPCR. Detection of H3K14ac levels at *GiIFS* (*Gi1.9955*) (B), *GiCYP73A* (*Gi2.1616*) (C), *GiPAL* (*Gi3.125*) (D), *GiPAL* (*Gi5.1238*) (E) and *GiCHS* (*Gi5.966*) (F) loci in 7-day-old *G. inflata* seedlings treated with SAHA or DMSO (control) for 6 hours and 24 hours by ChIP‐qPCR assay. Values represent means ± SD. Student's *t*-test,*P < 0.05, ** P < 0.01, *** P < 0.001, n = 3. Figure S11. SAHA treatment increased the levels of H3K27ac at the promoters of genes involved in flavonoid biosynthesis. (A) Schematic diagram illustrates the location of primers utilized for ChIP-qPCR. Detection of H3K14ac levels at *GiIFS* (*Gi1.9955*) (B), *GiCYP73A* (*Gi2.1616*) (C), *GiPAL* (*Gi3.125*) (D), *GiPAL* (*Gi5.1238*) (E) and *GiCHS* (*Gi5.966*) (F) loci in 7-day-old *G. inflata* seedlings treated with SAHA or DMSO (control) for 6 hours and 24 hours by ChIP‐qPCR assay. Values represent means ± SD. Student's *t*-test,*P < 0.05, ** P < 0.01, *** P < 0.001, n = 3. Figure S11. SAHA treatment increased the levels of H3K27ac at the promoters of genes involved in flavonoid biosynthesis. (A) Schematic diagram illustrates the location of primers utilized for ChIP-qPCR. Detection of H3K14ac levels at *GiIFS* (*Gi1.9955*) (B), *GiCYP73A* (*Gi2.1616*) (C), *GiPAL* (*Gi3.125*) (D), *GiPAL*(*Gi5.1238*) (E) and *GiCHS* (*Gi5.966*) (F) loci in 7-day-old *G. inflata* seedlings treated with SAHA or DMSO (control) for 6 hours and 24 hours by ChIP‐qPCR assay. Values represent means ± SD. Student's *t*-test, *P < 0.05, ** P < 0.01, *** P < 0.001, n = 3. Figure S12. Phylogenetic trees of AtHDACs and GiHDACs. A multiple sequence alignment of full-length amino acid sequences from GiHDAC genes was performed using ClustalX 2.0. The phylogenetic tree was constructed employing the Maximum Likelihood method in MEGA7.0. The branches of each subgroup are depicted in distinct colors. Figure S13. Chromosomal location of *GiHDAC* genes in *G. inflata* genome. The y axis represents the chromosome length in megabase pairs (Mbp). Figure S14. Environmental stimuli influenced histone acetylation status in *G. inflata*. Immunoblot analysis of histone acetylation in *G. inflata* seedlings subjected to NaCl, NaHCO_3_ (pH 8.0), cold, MeJA, and ABA treatments. Antibodies for H3ac, H3K9ac, H3K14ac, and H3K27ac were employed to assess histone acetylation levels, while an H3 antibody was used to determine loading quantity. Figure S15. (A) The morphological response of *G. inflata* seedlings to various environmental stressors. Seven-day-old *G. inflata* seedlings were subjected to treatments including ethanol (control), 250 mM NaCl, NaHCO_3_ (pH 8.0), cold (4 ℃), 100 μM MeJA, and 100 μM ABA for 7 days. Scale bar = 2.0 cm. (B) The fresh weight of *G. inflata* seedling roots under different treatments. The different lowercase letters indicate a statistically significant difference in acetylation modification levels at *P* < 0.05. Student's *t*-test, n ≥ 3. Figure S16. (A) Expression patterns of *GiHDAC* genes across various tissues and (B) in response to MeJA treatment. The HDAC genes are labeled on the right. The color scale values represent log_2_ (FPKM): red and green indicate high and low levels of transcript abundance, respectively. Tissue types and developmental stages of *G. inflata* are denoted at the bottom of the column. R1, one-year-old roots; R2, two-year-old roots; R3, three-year-old roots; L1, one-year-old leaves; L2, two-year-old leaves; L3, three-year-old leaves. Figure S17. Identification of OE-*GiHDA2b* and RNAi-*GiHDA2b* transgenic hairy roots. (A)PCR identification of OE-*GiHDA2b* and RNAi-*GiHDA2b* hairy roots. M denotes DNA marker. DNA from MSU440 strain carrying pCambia1300-*GiHDA2b* vector served as positive control ("+"). A seedling without transgene served as negative control ("-"). The expected PCR product sizes for OE-*GiHDA2b*, RNAi-*GiHDA2b*, and *rolB* were 1541 bp, 420 bp, and 738 bp, respectively. (B)Western blot identification of OE-*GiHDA2b* hairy roots. M indicates protein marker. The expected size of the GiHDA2b-GFP fusion protein was 70 KDa. Figure S18. Full scan (uncropped) western blots from supplementary figures. Uncropped full scans of western blots corresponding to the cropped versions presented within the supplementary figures. Figure S19. (A) The phenotype of one-month-old WT, OE-*GiHDA2b*,and RNAi-*GiHDA2b* transgenic hairy roots (scale bars: 2.0 cm). (B) Fresh weight measurements of the WT, OE-*GiHDA2b*, and RNAi-*GiHDA2b* transgenic hairy roots. Different lowercase letters indicate significant differences in acetylation modification levels at *P* < 0.05 among samples. Student's *t*-test, n ≥ 3. Figure S20. The schematic diagram shows the location of the primers used for ChIP-qPCR. Figure S21. Quantification of H3K18ac levels at *GiPAL* (*Gi3.125*), *GiCHS* (*Gi5.966*), *GiCHS4* (*Gi6.1582*), *GiCHR* (*Gi6.570*), *GiCHI* (*Gi2.1787*), *GiF2H2* (*Gi1.11009*), *GiLMT* (*Gi2.258*), and *GiPT* (*Gi7.1190*) loci in *GiHDA2b* transgenic hairy roots using ChIP‐qPCR assay. Values represent means ± SD. Different lowercase letters indicate significant differences in acetylation modification levels at *P* < 0.05. Student's *t*-test, n ≥ 3. Figure S22. qRT-PCR verification of expression patterns of 8 genes related to LCA biosynthesis in *GiHDA2b* transgenic hairy roots.The expression patterns of 8 genes were analyzed using qRT-PCR. *GiCOPS3* served as the internal control. Values represent means ± SD. Different lowercase letters indicate significant differences in acetylation modification levels at *P*< 0.05 among samples. Statistical analysis was performed using the Student's *t*-test, with n ≥ 3. 

## Data Availability

The datasets used and/or analyzed during the present study are available from the corresponding author on reasonable request.

## Abbreviations

NaB: Sodium butyrate
TSA: Trichostatin A
SIR: Sirtinol
SAHA: Suberoylanilide hydroxamic acid
LCA: Licochalcone A
LCC: Licochalcone C
HAT: Histone acetylase
HDAC: Histone deacetylase
LMT: Licodione 2’-*O*-methyltransferase
PAL: Phenylalnine ammonialyase
4CL: 4-Coumaric acid-CoA ligase
F3H: Flavanone-3-hydroxylase
COMT: Caffeate *O*-methyltransferase
IFS: Isoflavone synthase
ChIP: Chromatin immunoprecipitation
DEGs: Differentially expressed genes
PCA: Principal Component Analysis
